# The Application of Fungi and Their Secondary Metabolites in Aquaculture

**DOI:** 10.3390/jof10100711

**Published:** 2024-10-11

**Authors:** Abigail John Onomu, Grace Emily Okuthe

**Affiliations:** Department of Biological & Environmental Sciences, Walter Sisulu University, Mthatha 5117, South Africa; gokuthe@wsu.ac.za

**Keywords:** *Aspergillus niger*, mushroom, yeast, bioremediation, bioflocculation, buoyancy, probiotics, feed additive, antinutritional factors

## Abstract

Ensuring sustainability has increasingly become a significant concern not only in aquaculture but in the general agrifood sector. Therefore, it is imperative to investigate pathways to feed substitutes/best practices to enhance aquaculture sustainability. The application of fungi in aquaculture provides innovative methods to enhance the sustainability and productivity of aquaculture. Fungi play numerous roles in aquaculture, including growth, immunity enhancement and disease resistance. They also play a role in bioremediation of waste and bioflocculation. The application of fungi improves the suitability and utilization of terrestrial plant ingredients in aquaculture by reducing the fibre fractions and anti-nutritional factors and increasing the nutrients and mineral contents of plant ingredients. Fungi are good flotation agents and can enhance the buoyancy of aquafeed. Pigments from fungi enhance the colouration of fish fillets, making them more attractive to consumers. This paper, via the relevant literature, explores the multifaceted roles of fungi in aquaculture, emphasizing their potential to transform aquaculture through environmentally friendly and sustainable techniques. The effectiveness of fungi in reducing fibre fractions and enhancing nutrient availability is influenced by the duration of fermentation and the dosage administered, which may differ for various feed ingredients, making it difficult for most aquaculture farmers to apply fungi approximately. Therefore, the most effective dosage and fermentation duration for each feed ingredient should be investigated.

## 1. Introduction

Aquaculture is a significant contributor to global food and nutrition security and is expected to grow to meet the increasing demand of a growing population and economy [1]. Sustainable aquaculture practices are needed for the envisaged aquaculture growth to be achieved. These practices are essential not only for economic viability but also for environmental protection, sustenance and social responsibility [1]. Some aquaculture practices that require innovation and the adoption of sustainable practices include the over-reliance on fishmeal in feed production and the application of antibiotics and chemicals in preventing and treating disease [2]. The enhancement of the nutrient utilization of feed to obtain high feed efficiency and animal growth, the maintenance of the water quality, and wastewater/effluent bioremediation are also essential [3].

Due to the need for sustainable practices, alternative feed ingredients, such as terrestrial plants, are being incorporated into aquatic animal feed to reduce reliance on fish protein, minimize the cost of production and enhance aquaculture sustainability [4]. However, there are limitations associated with the use of terrestrial plants in aquafeed formulation. Generally, terrestrial plants are low in protein, high in fibre fractions (crude fibre, lignin, cellulose and hemicellulose) and contain antinutritional factors, which hinder their digestibility [5,6,7]. They are rich in phosphorus, which is essential for growth. However, the phosphorous in terrestrial plants is only available as phytic acid, which, unfortunately, aquatic animals cannot utilize [8,9]. Hence, when feeds containing terrestrial plants are fed to aquatic animals, the unutilized phytic acid is released into the culture system via faeces from the animals and uneaten feed. The water quality deteriorates on accumulation in the system [10]. The untreated effluent from such a system is detrimental to the aquatic ecosystem, which is the final destination of wastewater and effluent from aquaculture [11].

Aquatic farmers in developing countries cannot afford floating/extruded aquafeed. If they do, it impacts the cost of the final product, which, most times, may not be affordable to people within the locality [12]. Due to this, they use sinking feed or produce feed unconventionally using locally available ingredients [12]. The feed produced via this method tends to sink quickly on reaching water, making a large proportion of the feed unutilized by fish. Underutilization of feed and the nutrients present in the feed result in unmaximized growth and deterioration of the quality of the water, which then acts as a breeding ground for disease. Antibiotics and chemicals have been used to treat and prevent disease. Nevertheless, their use is discouraged due to the health implications on consumers, with the possibility that it might result in antibiotic resistance and detriment to the ecosystem [2]. Therefore, a sustainable substitute to prevent and control diseases is required.

Fungi are either unicellular or multicellular/filamentous and belong to the kingdom of fungi. They are ubiquitous eukaryotic organisms and heterotrophs. They have been exploited to produce a wide range of industrial products, such as fermented drinks and food, food additives, pigments, biofuels, enzymes, antibiotics, vitamins, fatty acids and sterols [13]. Fungi are important microorganisms with the potential to enhance aquaculture sustainability. The application of fungi in fermenting plant ingredients (terrestrial protein sources) reduces the antinutritional factors and fibre fractions, such as hemicellulose and lignin [7,14,15]. It also increases the nutrient availability in plant ingredients, making them better suitable for use as aquatic animal feed. For example, fermentation with fungi increases the protein content and reduces the fat content of plant protein [15,16,17]. The reduction of fat content in plant protein is suitable for aquatic animals as a high-fat content is not required in their diet [18]. Fungi are efficient bioremediators and have been applied in the bioremediation of wastewater. They can also remove heavy metals from wastewater and minimize the effect of heavy metal pollution on aquatic animals [14,19]. Fungi, such as yeast (*saccharomyces cerevisiae*) and mould (*Aspergillus niger*) possess a flotation ability and can be used when enhancing the buoyancy of aquafeed, thus improving feed efficiency and utilization [20,21]. Fungi also serve as flocculants and are efficient in the harvesting of microalgae, thus minimizing the cost involved in microalgae harvesting [22,23]. The potential benefits of fungi are numerous, including enhanced productivity and a more efficient, sustainable and environmental practice contributing to improving aquaculture sustainability. However, these benefits need to be organized in the literature in order to create awareness and promote knowledge for its adoption. This is because research has revealed that the potential of fungi has not been adequately utilized/exploited in aquaculture practice due to various factors. Therefore, this study seeks to investigate significant findings on its application in aquaculture.

### 1.1. Objectives

The objective of this study is to expose the application of fungi in aquaculture and the limitations associated with its application. The study aims to create awareness of fungi and its beneficial use in aquaculture.

### 1.2. The Description of Fungi

Over 3.8 million species of fungi exist in nature; however, a large fraction are unidentified [24]. Fungi are categorized into eight phyla, which are Ascomycota, Basidiomycota, Blastocladiomycota, Chytridiomycota, Glomeromycota, Microsporidia, Neocallimastigomycota and Zygomycota. Fungi are either saprobes, living in decomposing organic matter, or parasites; they possess cell walls composed of chitin and polysaccharides. They are immotile and reproduce via spores [25]. Examples of fungi of importance to aquaculture are mould, yeast and mushroom. Some fungi species are pathogenic, causing mild or severe diseases in plants, animals and humans [26]. However, some are of economic importance. For example, yeast metabolism is exploited to produce a wide range of industrial products such as fermented drinks, food additives, pigments, biofuels, enzymes, antibiotics, vitamins, fatty acids and sterols [13].

Moulds are filamentous fungi found everywhere, including in soils, compost, litter and decaying plant matter [24]. Mould grows on organic matter in the presence of oxygen (aerobically). The most common mould used in aquaculture is *A. niger* and *A. oryzae*, which are generally non-pathogenic. The enzymes from *A. niger* are generally regarded as safe (GRAS) for consumption/food production [27]. Yeasts are microscopic, unicellular, eukaryotic organisms in the fungi kingdom [28,29]. Yeasts are facultative anaerobes, implying that they can survive, grow and thrive with or without oxygen [30]. They are found everywhere in the environment, possessing nuclear membranes and cell walls but no chloroplast [28]. They are heterotrophs and obtain their nutrients and energy from live and dead organic matter [31]. Nutrition by yeast is attained via the production and secretion of glycolytic, proteolytic and lipolytic enzymes, which digest organic matter. Alternatively, nutrition is obtained by absorbing amino acids and monosaccharides via their cell wall [28]. Some yeast genera are disease-causing, while some greatly benefit man. For example, the genera *Cryptococcus*, *Trichosporon*, *Candida* and *Torulosis* are pathogenic, while the species *Candida utilis*, *S. cerevisiae* and *Kluyveromyces marxianus* are of benefit to humans and animals [32,33].

Mushrooms are higher fungi, classified as Ascomycetes (*Morchella*, *Tuber* etc.) and Basidiomycetes (*Auricularia*, *Tremella*, *Agaricus* etc.). Mushrooms are categorised into edible mushrooms and medicinal mushrooms. Medicinal mushrooms are only suitable as medicine and not as food [34]. The bioactive compounds in mushrooms include dietary fibres, proteins, amino acids, alkaloids, oligosaccharides, polysaccharides, alcohols, vitamins and minerals [35]. Various species of mushrooms are used in aquaculture, including *Ganoderma* sp., *Pleurotus* sp. (Oyster mushroom), *Lentinula edodes* (Shiitake mushroom), *Coriolus* sp. (Turkey tail mushroom), *Cordyceps* sp. (Caterpillar mushroom) and *Agaricus bisporus* (White button mushroom). The stalk, mycelia, polysaccharides, extracts and dry powder derived from mushrooms are applied in aquaculture [36].

## 2. Application of Fungi and Its Metabolites in Aquaculture

### 2.1. Source of Antibiotics, Probiotics and Prebiotics

Aquatic environments are complex ecosystems characterised by diverse microorganisms (including beneficial and pathogenic microorganisms) which influence the health of cultured animals [37]. The traditional method employed in curbing aquatic pathogens is the application of antibiotics and chemicals, which may lead to antibiotic resistance or even negatively impact the aquatic ecosystem [2]. Fungi generate a variety of bioactive substances, such as antibiotics, enzymes and secondary metabolites, which can hinder bacteria, and viruses and pathogenic fungi growth [38]. Fungi, such as *Penicillium* and *Aspergilus* sp., produce penicillin and other antibiotics with broad-spectrum antibacterial activity [39]. *Trichoderma asperellum* MT02, isolated from sponges, exhibited antibacterial activity against *V. harveyi* and *V. alginolyticus*, pathogens of white leg shrimp (*Litopenaeus vannamei*) known to result in disease leading to a substantial economic loss [40]. Similarly, fungi metabolites of marine red alga *Nodulisporium* sp. KT29 possess antibacterial compounds that inhibit bacteria pathogens such as *Aeromonas salmonicida*, *Vibrio anguillarum*, *V. harveyi* and *Yersinia ruckeri* [38]. 

**Figure 1:**
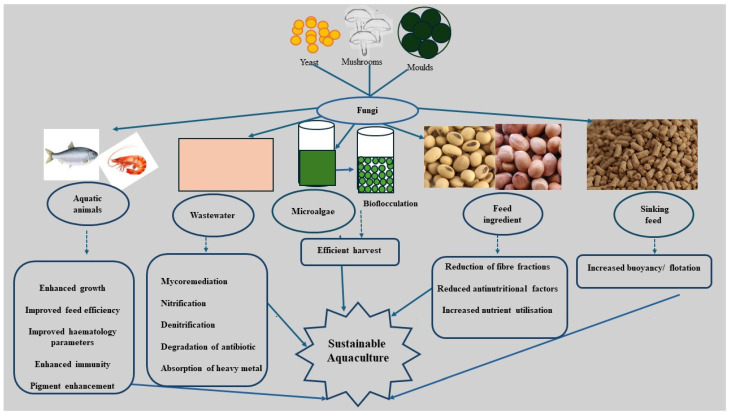
The potential application of fungi in aquaculture.

Fungi also function as probiotics/prebiotics when supplemented in diets, enhancing growth, feed efficiency, immune and haematology parameters and disease resistance against pathogens [41] (Figure 1). Probiotics can be defined as “a live microbial adjunct which has a beneficial effect on the host by modifying the host-associated or ambient microbial community, ensuring improved use of the feed enhancing the nutritional value of the feed and enhancing the host response towards disease or improving the quality of its ambient environment” [42]. Prebiotics are indigestible feed components that improve health by promoting the growth and metabolism of beneficial bacteria in the gut [43]. *Nodulisporium* sp. KT29 metabolite, when incorporated into the feed of *Litopenaeus vannamei,* resulted in a higher survival rate, growth rate and better feed efficiency ratio (FCR) within 21 days, when compared with the control (without fungi supplementation) [44]. The improved growth, survival, and FCR displayed by shrimp-fed diets containing fungi metabolites are reportedly due to the phytochemicals (β-glucans and polyphenols) contained in the metabolites, which act as immunostimulants and antioxidants [44]. Fermented mushroom (*Pleurotus ostreatus*) added to the diet of juvenile Amur catfish, *Silurus asotus,* at 0.1% and 0.2% concentrations for eight weeks showed a significantly higher weight gain and specific growth rate than that of the control [45]. *Aspergillus niger*, when fed to common carp *Cyprinus carpio* for 60 days at a concentration of 10^6^ in the diet, resulted in improved length, weight, protein efficiency ratio, lipid efficiency ratio and lower FCR than the control. Similarly, carp fed *A. niger*-containing diets, at a concentration of 10^6^, outperformed those with a concentration of 10^3^ in all indices mentioned above [46] (Table 1). Likewise, Pacific white shrimp (*Penaeus vannamei*) fed diets supplemented with a 1.5 g/kg diet of *A. niger* had higher weight gain, length and specific growth rate than the control and those fed 3 g/kg of *A. niger* [47].

Fungi supplementation in diets also plays immunostimulatory roles in aquatic animals, resulting in enhanced immunity and haematology parameters, which are good health indicators [48]. *Litopeneaus vannamei* fed diets containing *Nodulisporium* sp. KT29 metabolite for 21 days exhibited a higher survival rate than the control on exposure to *V. harveyi* pathogen. Ten days after being challenged with the pathogen, shrimp-fed diets containing the metabolite had higher phenoloxidase and respiratory burst activity than the control. In contrast, shrimp in the control group had higher haemolymph glucose levels compared with those fed diets containing metabolites. This indicates that shrimp fed fungi-supplemented diets had a higher immunity compared with those fed the unsupplemented diets [44] (Table 1).

Fish fed fungi diets reportedly had a higher fungi cell load in their gut than those without fungi. However, this was influenced by the concentration of fungi in the feed, such that the fungi load increases with the increase in fungi content of the feed [46]. In common carp, *C. carpio* fed diets containing 10^6^ and 10^3^ *A. niger*, immunological parameters (plasma lysozyme activity and total immunoglobulins levels) and haematology indices (red blood cells, haemoglobin count, mean corpuscular haemoglobin, white blood cell count, mean corpuscular volume and lymphocyte) increased after 60 days compared with the control. However, fish fed diets containing 10^6^ *A. niger* had higher white blood cell counts and haematocrit compared with the control and fish fed 10^3^ *A. niger* diet [46]. Nitroblue tetrazolium, antiprotease and lysozyme activity of *Labeo rohita* was also shown to increase with an increase in spent mushroom substrate (*Cordyceps militaris*) in diets [49]. Cheng et al. used four experimental diets containing 0, 0.5, 1 and 1.5 g/kg of *A. niger* in diets fed to white shrimp, *P. vannamei* for 56 days [50]. Fish fed 1 and 1.5 g/kg of *A. niger* had higher resistance to *V. parahaemolyticus* infection, significantly lower mortality rates to *V. parahaemolyticus* and higher lysozyme activities than the control. On assessing the vibrio-like bacteria counts in the gut of the shrimp, shrimp fed *A. niger*-containing diets had lower vibrio-like counts than the control. Additionally, the phagocytic and phenoloxidase activity in the haemocyte of fish fed the 1 and 1.5 g/kg of *A. niger* were higher than those of the 0.5 g/kg and control groups. Zhang et al. used 0, 1.5 and 3 g of *A. niger* per kg of feed on Pacific white shrimp (*P. vannamei*) for a month [47]. Catalase, acid phosphatase, alkaline phosphatase, superoxide dismutase (SOD) and phenol oxidase were higher in fish fed fungi diets than those of the control, indicating a higher immunity (Table 1). Sarlin and Philip investigated marine yeasts *Debaryomyces hansenii* (S8) and *Candida tropicalis* (S186) as immunostimulants for *Fenneropenaeus indicus*, comparing their efficacy with that of *Saccharomyces cerevisiae* S36 [51]. After feeding the shrimps for two weeks, with diets containing these fungi, they were orally challenged with white spot syndrome virus (WSSV), a highly pathogenic virus causing 100% mortality in aquatic animals within 3–7 days. Shrimp fed the various diets showed a similar reduction in haemocyte count within two days after being challenged with the virus. However, shrimp fed with the two marine fungi had a higher survival rate (50%), phenoloxidase and nitroblue tetrazolium activity than those fed *S. cerevisiae* and the control. The marine yeast performed better than *S. cerevisiae*, probably because they had a higher content of β-1,3 glucan [51]. 

**Table 1:** The application of fungi in aquatic animals and their effect on growth, haematological parameters and disease resistance.

Fungi	Fungi Species	Species	Concentration and Duration	Effect on Growth and Survival	Haematological Parameters	Disease Resistance	Reference
Mushroom (powder)	*Pleurotus eryngii*	Koi carp fingerlings (*Cyprinus carpio* koi)	0, 0.5, 1, 1.5 and 2% for 61 days	FW, WG, SGR, (↑) (1.5%). FCR (↓) (0.5, 1, 1.5 and 2%) SR (↑) (0, 0.5, 1, 1.5 and 2%)	WBC, Hb, Ht, MCV, MCH, monocyte, MCHC (↑) (1.5 and 2%) Digestive enzymes (Trypsin and lipase) (↑) (2%) α-amylase (↑) (1, 1.5, 2%)	NA	[52]
	*Ganoderma lucidum*	Nile Tilapia (*Oreochromis niloticus*)	0, 0.5, 1, and 2% for 90 days	FW, WG (%), SGR (↑) (1%) FCR (↓) (10%) SR (↔) (0, 0.5, 1, and 2%)	NA	NA	[53]
Mushroom (fermented by product)	*Pleurotus ostreatus*	Amur catfish (*Silurus asotus*)	0, 0.1, 0.2, 0.4, 0.8% for 56 days.	WG, SGR (%) (↑) (0.1 and 0.2%) FE, PER (↑) (0.2)	Haematocrit (↑) (0.2%) Lysozyme activity (↑) (0.1%) PCV (↑) (0.2, 0.4 and 0.8%)	NA	[45]
Spent mushroom substrate	*Cordyceps militaris*	*Labeo rohita*	0, 1, 2, and 3% for 60 days.	NA	NBT, antiprotease and lysozyme activity (↑) (1, 2 and 3%)	SR (↑) after challenged with *A. hydrophila* (1, 2, and 3%).	[49]
Mould	*Aspergillus niger*	*Penaeus vannamei*	0, 0.15 and 0.3% for 28 days	WG (%), SGR (%) and length gain (%) (↑) (1.5%)	ACP and AKP (↑) (1.5%) SOD and catalase activity (↑) (1.5 and 3%) Lysozyme activity (↓) (1 and 3%)	NA	[47]
	*Aspergillus niger*	Common Carp (*Cyprinus carpio*)	0, 1 × 10^3^ and 1 × 10^6^ for 60 days	FW, WG, FL, PER, LER (↑) (10^3^ and 10^6^). FCR (↓) (10^3^ and 10^6^).	Lysozyme activity, Ig, RBC, Hb (↑) (10^3^ and 10^6^) Body protein and dry matter composition (↑) (10^3^ and 10^6^). Body lipid composition (↓) (10^3^ and 10^6^). Amylase and lipase digestive enzymes (↑) (10^3^ and 10^6^).	N/A	[46]
	*Aspergillus niger*	White shrimp (*Penaeus vannamei*)	0, 0.5, 1 and 1.5 g (kg^−1^ diet) for 56 days	FW and WG (↑) (0.5, 1 and 1.5 g kg^−1^ diet) FCR (↔) (0.5, 1 and 1.5 g kg^−1^ diet)	Lysozyme, phygocytic and phenoloxidase activity (↑) 0.5, 1 and 1.5 g kg^−1^ diet) Reduced vibrio-like count in the gut of the fish	Mortality (↓) (0.5, 1 and 1.5 g kg^−1^ diet)	[50]
Yeast	*Saccharomyces cerevisiae*	Nile Tilapia *Oreochromis niloticus*	0, 1, 2 and 4 g kg^−1^ for 60 days.	SGR, PER (↑) (4 g kg^−1^)	gut villus wall thickness, villus length, width, and area (↑) (4 g kg^−1^)	N/A	[54]
	*Saccharomyces cerevisiae*	Nile Tilapia *Oreochromis niloticus*	0, 3, 5 and 7% for 84 days	WG (↔) PER (↑) (3, 5 and 7%) FCR (↓) (3, 5 and 7%)		Mortality (↓) (7%)	[55]
	*Saccharomyces cerevisiae*	Snakehead *Channa punctatus*	0 and 2.5 g kg^−1^ for 56 days.	WG, PER, FCR, (↔) SGR (↑)	Intestinal protease, lipase, and amylase enzyme activities (↑)	Mortality (↓)	[56]

Note: (↑)—significant increase, (↓)—significant decrease, (↔)—no significant difference. WBC—white blood cells, Hb—haemoglobin, Ht—haematocrit, MCV(fl)—mean corpuscular haemoglobin volume, MCH—mean corpuscular haemoglobin, MCHC—mean corpuscular haemoglobin concentration (MCHC), SR—survival rate, FW—final weight; WG—weight gain; SGR—specific weight gain, PER—protein efficiency ratio, LER—lipid efficiency ratio, NBT—nitroblue tetrazolium, ACP—acid phosphatase activity, AKP—alkaline phosphatase activity, Ig—immunoglobins. Variation in treatments compared with the control.

### 2.2. Reduction of Anti-Nutritional Factors and Fibre Fractions in Plant Ingredients

The use of alternative protein sources, such as protein from terrestrial plants for aquafeed production, is encouraged to reduce reliance on fish meal and increase the sustainability of aquaculture [2]. However, the antinutrients naturally present in terrestrial plant proteins are a limiting factor to their use. Antinutrients are substances which directly or indirectly (through metabolic substances produced in living organisms) interfere with feed utilization and negatively impact animal health and productivity [57]. Some antinutrients associated with terrestrial plant proteins include tannin, saponins, lectins, gossypol, glucosinolates, protease and amylase inhibitors. Tannin has a bitter taste that limits acceptability, palatability, feed ingestion and growth in aquatic animals [58]. Glucosinolates cause thyroid dysfunction, which may affect metabolism and growth, while saponins inhibit active nutrient transport, cause damage to the respiratory epithelium of the gills and inhibit growth. 

The application of fungi in terrestrial plants reduces the antinutritional factors in terrestrial plants, making them more suitable for utilization in the production of aquafeeds. Fungi are mostly incorporated as a fermentation agent. The fermentation of groundnut oil cake with *A. niger* led to the reduction of saponin, phytic acid and tannin by 49%, 37.57% and 83.60%, respectively [59]. Similarly, the fermentation of rapeseed with *A. niger* led to a 76.89% reduction in glucosinolates [60]. Natural fermentation without fungi can reduce some anti-nutritional factors to a degree, but not as efficiently as fungi. For example, the natural fermentation of soya bean meal reduced trypsin content in SBM by 63.85%. However, fermentation with yeast (*S. cerevisiae*) and *A. niger* reduced the trypsin content in SBM by 92.47 and 94%, respectively. Additionally, trypsin inhibitor was totally removed by fermenting guar meal with *A. niger* and *S. cerevisiae* [59]. 

The application of plant ingredients in fish diets increases the fibre content of the diets. A high fibre fraction in feed reduces feed digestibility as most aquatic species cannot utilize fibre. Fungi are beneficial in reducing the fibrous content of plant-based diets, thus increasing digestibility. This is achieved by the release of fibre hydrolytic enzymes such as xylanase, pectinase and cellulase when applied in solid-state fermentation [60,61,62,63]. The reduction of fibre content during fermentation could also be due to microbial digestion, breaking down components like cellulose and hemicellulose, which are undigestible by fish, into simple sugars [64]. Fermentation of rape seed cake with *A. niger* reduced the neutral detergent fibre by 9.17% [60] (Table 2). Fermentation of soyabean meal, rapeseed meal, groundnut oil cake, sunflower oil cake, and guar meal with *A. niger* and yeast (*S. cerevisiae*) resulted in reduced fibre fractions (neutral detergent fibre (NDF), acid detergent fibre (ADF), cellulose, hemicellulose and lignin) compared with natural fermentation and bacteria fermentation with *Lactobacillus acidophilus* (Table 2). Fermentation of cassava peel with *A. niger* significantly reduces the fibre content compared with those fermented naturally. The digestibility of cassava peel increased from 11.74% to 48.73% after fermentation [65]. *Oreochromis niloticus* (Tilapia) fed diets containing unfermented cassava peel and fermented cassava peel showed similar feed consumption and feed efficiency, demonstrating that *A. niger* in diets was well tolerated and accepted. Additionally, *O. niloticus* fed diets containing fermented cassava peel had higher dry matter, protein and energy digestibility and daily growth rate than fish fed diets containing unfermented cassava. However, fat digestibility was higher in fish fed the unfermented cassava peel than those fed the fermented cassava peel [65]. *Trametes coccinea* (i.e., *Pycnoporus coccineus*, scarlet bracket-fungus mushroom), *Lentinula edodes* (shiitake mushroom), or *Pleurotus sajor-caju* (Indian Oyster mushroom) were used to ferment rapeseed meal (RM), soya bean meal (SBM), algal meal and macrophyte meal. Fermentation with these fungi reduced the NDF of all feed ingredients except for the macrophyte meal. The digestibility of phosphorous, manganese, zinc and copper significantly increased in rapeseed fermented with *T. coccinea* or *L. edodes* [17].

The reduction of fibre via fungi fermentation is influenced by the fungi dosage and the duration of fermentation. The reduction of crude fibre and its fraction contents (hemicellulose, cellulose, ADF, NDF) was higher when rice bran was fermented with 2 g of *A. niger* per 100 g, compared with 0.5, 1, 1.5 g and the control (unfermented rice bran). However, with rice bran fermented with 2 g of *A. niger*, the reduction in crude fibre increased with an increase in fermentation time; those with a 48-hr fermentation period had the lowest crude fibre content [65] (Table 2).

### 2.3. Increased Nutrient Availability in Feed

Fermentation of aquaculture feed ingredients/feed with fungi increases nutrient content such as protein and amino acids, making the feed/feed ingredient more suitable for aquatic animals [20]. Fermentation of groundnut oil cake (GNC) using *A. niger* led to about a 21% increase in protein content [59]. Fermentation with *A. niger* also enhanced the protein and ether extract content of rapeseed [15]. *Trametes coccinea*, *L. edodes*, or *P. sajor-cajuin* increased the crude protein content of rapeseed meal, soya bean meal (SBM), algal meal and macrophyte meal upon fermentation [17]. Groundnut oil cake fermented with *A. niger* also had increased essential amino acid content. The methionine and lysine content of fermented GNC had a 50% increase compared with the unfermented GNC. Similarly, histidine, tryptophan and threonine increased by about 20% upon fermentation with *A. niger* [20,59]. Hariyono et al. have also reported that fermentation of pellet with *Rhizopus* sp. led to an increase in the amino acid content of the pellet [20]. Fermentation of aquafeed pellets with *Rhizopus* sp. inoculum at a dosage of 1%, 2% and 3% for 30, 40 and 50 h reduced the fat content of the pellet. However, the reduction of fat content was affected by the dosage of inoculum and fermentation duration such that pellets fermented with 3% inoculum for 50 h had the lowest fat content [20]. The fat reduction in pellets by *Rhizopus* sp. has been attributed to the production of lipase enzymes by *Rhizopus* sp. during fermentation. The lipase enzyme hydrolyses triacylglycerol into free fatty acids. The fungi use fatty acids as energy for growth during fermentation, leading to reduced lipid content [20].

Fermentation with fungi seems to be more efficient in enhancing the nutrient composition of feed ingredients than fermentation with bacteria. Fermentation of sargassum powder with *A. niger*, *S. cerevisiae* and *Lactobacillus* spp. increased the protein content of sargassum powder and reduced lipid and carbohydrate content, unlike the control (unfermented sargassum powder). However, fermentation with *A. niger* led to a significantly higher protein (from 7.5 to 10.1%) and reduced carbohydrate content (50.7% to 29.1%) than those of *S. cerevisiae* and *Lactobacillus* spp. While fermentation with *S. cerevisiae* reduced lipid content (1.33% to 0.43%) significantly compared with *A. niger* and *Lactobacillus* spp. [74]. 

Phytase enzymes obtained from fungi can also be used to enhance the availability of some minerals [75,76] and have been employed to improve dietary mineral retention in aquatic animals [77,78]. Channel catfish fed phytase-containing diets had a significantly higher concentration of manganese, ash, calcium and phosphorous deposited in the bones than fish without phytase supplementation. The phytase concentration also influenced the deposition of these minerals in the bone of the fish. For example, the magnesium content of the bone was similar for fish fed 500 U/kg and for the control. However, fish fed 1000 U/kg and above of phytase had significantly higher bone magnesium than the control [79]. This implies a higher nutrient availability in fish fed diets containing a higher phytase concentration.

### 2.4. Increased Mineral Utilization and Reduced Nutrients in Effluents

Phosphorous is one of the essential minerals required for growth in aquatic animals [80]. Phosphorous is naturally found in feed ingredients of plant origin. It is present as phytic acid (myo-inositol 1,2,3,4,5,6-hexakisphosphate or phytate) and makes up about 80% of the total phosphorous in plant ingredients [81]. Aquatic animals, especially fish, cannot utilize the phytic acid in these plant ingredients. This is because most aquatic animals lack or have low amounts of phytase enzyme, a requisite for the utilization of phosphorous bound to phytic acid [82,83]. Phosphorous deficiency and suboptimal levels of dietary phosphorous in diets of aquatic animals result in reduced growth performance, operculum and vertebral deformities, poor bone mineralisation, and reduced immunity [84,85]. This necessitates the supplementation of phosphorous during feed formulation. Inorganic phosphorous, such as dicalcium phosphate or monocalcium phosphate, are usually added in aquafeed formulation. With the use of phosphorous supplements in various sectors, including the agricultural sector, it has been predicted that, by 2050, the world reserve will be depleted [86,87]. Additionally, excess use of phosphorous could be detrimental to the environment, causing pollution of water bodies and eutrophication.

Phytase enzymes can replace dicalcium phosphate without any detrimental effect on aquatic animals. Applying phytase enzymes can also reduce the adverse effects attributed to phytic acid, such as limitation of availability of trace minerals, like zinc; water quality deterioration; and environmental pollution [88,89]. The water quality deterioration and environmental pollution arise via the decomposition of uneaten feed and faeces rich in phytic acid. Phytic acid is released into the rearing system and converted to phosphorous after being acted upon by microorganisms. Therefore, phosphorous is released into the rearing system, deteriorating water quality [10]. Phosphorous-rich wastewater released into the environment adversely affects the environment. Phytase enzymes can be produced from plants, bacteria and fungi [90]. The limitation in the production of phytase enzymes from plants is that the synthesis methods are inefficient, time-consuming and expensive [90]. Fungal phytase, one of the metabolites produced by fungi, is more desirable than bacterial phytase because of its thermostability and more significant chitin effects. Bacterial phytase is limited by protease in the fish gut, high substrate specificity and acidic to alkaline pH activity [91].

Fish that were fed diets in which dicalcium phosphate was replaced with phytase enzyme from fungi had similar weight gain, FCR, and protein efficiency ratio to fish fed diets containing dicalcium phosphate [92,93]. Interestingly, adding phytase enzyme from fungi at 10 g/kg of feed led to higher phosphorous retention compared with fish fed diets containing dicalcium phosphate (15 g/kg) [92]. An advantage of using phytase of fungal origin in aquaculture is that it reduces the phosphorous load in the water and thus the environment, when compared with inorganic phosphorous, which could leach into the water column from feed. Phytase enzyme from fungi reduces the phytate content in feed ingredients [93]. The inclusion of phytase from *A. niger* in a diet degraded the phytate content of that diet during feed production. The efficiency of fungal phytase in reducing phytate is also dose dependent. Yan et al. (2002), have reported that a dose of 2000 U/kg and above in diets reduces phytate content when compared with a diet containing 500 U/kg of phytase. Similarly, Yan et al., (2002) formulated a 96% plant protein diet for channel catfish prepared with the inclusion of phytase from *A. niger* at varying concentrations in diets (0, 500, 1000, 2000, 4000 and 8000 U/kg diet). The analysis of phytate concentration in the stomach showed a lower concentration of phytate as early as 4 h after feeding for fish fed diets of 100 U/kg of phytase in their diet and at 12 h after feeding with 500 U/kg of a phytase-containing diet. Fungi phytase should be applied by spraying on formulated diets after extrusion, as temperature affects its efficacy and may not withstand the temperature of feed production and extrusion [93].

### 2.5. Fungi as a Buoyancy/Flotation Agent in Aquafeed

Artificial feeds used in aquaculture are categorised into floating (extruded) and sinking feed (non-extruded). These feeds are given to animals depending on their feeding habit in the water column [94]. Floating feeds are appropriate for animals that feed on the water’s surface while sinking feeds are suitable for animals that feed at the bottom [94]. Feed floats in water only when its density is lower than that of water [20]. Floating feeds are made by extrusion involving the application of heat. Thus, floating feeds are hydrophobic, making them highly stable in water [95]. They are, however, expensive, as a high investment cost is required in their production. Though sinking feeds are cheaper compared with their floating counterparts, they easily reach the bottom of the culture system [94]. On reaching the bottom of the culture system, they disintegrate, impacting the water quality negatively [96,97]. A high percentage of the sinking feeds introduced into the culture system are not utilized by fish, which is uneconomical. Generally, small-scale farmers in developing/countries may not be able to afford extruded feed; they end up buying sinking feeds or producing sinking feed with locally available ingredients [12]. They feed sinking feeds to their animals regardless of their feeding habits. Therefore, they encounter challenges such as water quality deterioration and reduced efficiencies from feed wastage [98]. 

Fungi have the ability to enhance the buoyancy/flotation of fish feed without the need for physical extrusion [20,21]. Flotation of feed via the use of fungi can be achieved when applied in solid-phase fermentation. The flotation of fungi fermented feed is imputed to the surface fungi mycelium (network of hyphae, which is cotton-like and whitish), preventing penetration of water into the poriferous spaces on the pellet. *Rhizopus* sp. and yeast are used to increase the flotation of feed [99]. A commercial sinking feed has been fermented using *Rhizopus* sp. [21]. The buoyancy and stability of the final product from fermentation were compared with those of commercial floating and sinking feed from the same producers. About 80% of the fermented feed remained afloat after immersion in water for 15 min. The fermented feed had a higher stability and buoyancy compared with the sinking feed counterpart. However, the commercial extruded feed had higher stability and buoyancy in water compared with *Rhizopus* sp. fermented feed [21]. In a study by [20], the experimental feed was fermented with three doses of *Rhizopus* sp. inoculum (1%, 2% and 3%) and three durations of fermentation (30, 40 and 50 h). All fermented pellets had enhanced buoyancy of feed compared with the control, which sank within 5 min of immersion in water. Feed fermented with 3% inoculum for 50 h had the highest buoyancy, with about 83% and 67% of the feed remaining afloat after an hour of immersion in non-aerated and aerated water, respectively. *Rhizopus* sp. inoculum fermentation also improved feed stability, with fermented feed resulting in higher stability than the control. Feed fermented with 3% inoculum for 50 h had 92.38% stability in unaerated water and 89.55% in aerated water when placed in water for 60 min [20]. Yeast (*S. cerevisiae*) has also been used as a flotation agent in aquafeed formulation [95,100]. [101] formulated experimental diets with four concentrations of yeast (5, 7.5, 10 and 12%) and four incubation periods (30, 40, 45 and 60 min) at a temperature of 38° C. All diets showed buoyancy when they were tested for it. However, those formulated with 7.5% yeast and incubated for 30 min had the best buoyancy, with 95% of the feed remaining afloat in water for 30 min.

Beyond buoyancy, the stability of the feed is significant in aquaculture. Water is known to disintegrate aquatic feed, therefore, during their formulation (either floating or sinking), binders are added to bind feed ingredients so as to enhance their stability [102,103]. The capacity of fungi to grow on a substrate of organic origin and also bind the substrate particles makes them suitable for use as biological binders in the manufacturing of aquafeed [99]. The binding effect achieved by fungi in aquafeed is attributed to the fungi hyphae (thread-like filament) penetrating thoroughly into the pellets, filling up spaces in the pellet and interlocking the feed ingredients. The various studies available on yeast have shown it to be a good flotation agent and not a binder. Thus, making *Rhizopus* sp. more beneficial as both a flotation and binding agent. Fermented feed is an alternative to sinking feed and can be applied in small-scale farming where extruded feed is not available or where the farmers produce their feed using locally available ingredients. 

### 2.6. Pigment

The colour and outward appearance of cultured animals play important roles in the attractiveness, value, demand and acceptance of a product [104,105] (Figure 1). Aquatic animals do not have the ability to synthesize their colour pigment. Therefore, the colour pigment has to be provided in the diet to impart a suitable colour. In the case of ornamental fishes, they generally lose their colouration due to the stress associated with captivity. They also lose their colour when fed diets lacking pigment [105]. Colour-enhancing feed additives are available in the form of carotenoids and pigments. Sources of carotenoids and pigments for fish may be synthetic or natural. Natural sources of pigments include vegetables and fruits such as red beetroot, carrot, okra, cabbage, and tomatoes. Synthetic pigments are characterized by high cost, poor uptake levels, and health and environmental concerns. These limitations of synthetic pigments have led to a focus on non-toxic pigments from natural sources [106]. However, pigments from natural sources, such as plants, are constrained by their limited availabilities, irregular harvest, land use, labour intensiveness and seasonal availability [107,108].

Plant pigments are preferred for use in the food industry, but, due to their limitations, research interest has shifted towards filamentous fungi with a history as a food source [109,110]. Astaxanthin, the primary carotenoid found in aquatic animals and which are orange-reddish, are usually added to the feed of salmon, lobster shrimps and trout for colour enhancement [111,112,113]. Astaxanthin is produced commercially from crustaceans, microalgae, bacteria and chemical synthesis [114,115]. Owing to its limited sources and reduced output, astaxanthin derived from natural sources is expensive [116]. Therefore, those derived from chemical synthesis are mainly used as a colour enhancer in aquaculture. Patronizing astaxanthin from synthetic sources in aquaculture encourages its production, which could be a source of pollution to the environment. Additionally, the use of synthetic astaxanthin in aquaculture may pose health risks to consumers [117].

Filamentous fungi (especially basidiomycetous [mushrooms], ascomycetous fungi and lichen) and yeast produce a diverse range of pigments, including flavins, phenazines, melanins, quinines and azaphilones [116,118,119]. The pigments obtained from fungi are secreted as secondary metabolites. They are produced as a reaction to unfavourable environmental conditions, such as high/low temperatures and excessive/prolonged exposure to light and stress, such as exposure to limited/insufficient availability of nutrients [120,121]. An advantage of pigment produced by fungi over pigments from other natural sources is that it does not make use of agricultural land meant for food production [108,110]. 

*Neurospora* spp. are among the fungi generally recognized as safe (GRAS), as they are not known to produce mycotoxins [122]. An advantage of *Neurospora* spp. as a pigment-producing fungi, is that it can be grown on a variety of substrates, including industrial residuals and lignocellulose [122]. Yeast species, such as *Kluyveromyces marxianus*, *Xanthophyllomyces dendrorhous* (formerly *Phaffoa rhodozyma*) and *Rhodotorula mucilaginosa*, also produce pigments [123]. Lin et al. incorporated astaxanthin from yeast (*Kluyveromyces marxianus*) and synthetic astaxanthin, Carophyll Pink and Lucanthin Pink, into the diet of white shrimp (*Penaeus vannamei*) [124]. Within four weeks of diet application, shrimp fed astaxanthin from yeast had increased redness values. Wang et al. also reported that the dietary inclusion of a red marine yeast *Rhodotorula mucilaginosa* JM-01 for six weeks resulted in increased pigmentation (redness and yellowness) of the body and shell astaxanthin content of the shrimp, *Penaeus vannamei* [125]. Marine yeast *Rhodotorula paludigena* VA 242, when incorporated into feed, improves the colouration of *Cyprino carpio* within 60 days of use [106]. Mushrooms mixed with cladoceran diets (*Pleurotus ostreatus* and *Pleurotus djamor*) reportedly improve the colouration of Siamese fighting fish (*Betta splendens*) when included in diets and when compared with the control, which had cladoceran powder alone [126]. Though fungi pigments are effective at enhancing the colouration of aquatic animals, a precaution in their use, especially with moulds, is to prevent the introduction of toxins/mycotoxins from the fungi. Some fungi-derived pigments are already commercialized, for example, canthaxanthin and ankaflavin pigments produced from the filamentous fungi *Monascus* sp. and Arpink redTM pigment produced from *Penicillium oxalicum* var. armeniaca [127].

### 2.7. Bioremediation

Water quality is of major importance in aquaculture, as aquatic animals depend on the water for life processes, which include survival, feeding, excretion and metabolic activities [2]. Excess nutrients in water emanating from unconsumed feed and excretory waste, including faeces, deteriorate water quality, leading to chemical and biological oxygen demand, which is detrimental to aquatic lives [96,97]. The presence of heavy metals and bacteria proliferation in water also pose a threat to aquatic animals. Aquaculture effluents are nutrient-dense, containing nutrients such as organic matter, phosphorus and carbon. The discharge of nutrient-dense effluent without treatment into the aquatic ecosystem is detrimental to the environment and lives within the environment [128]. This is one of the reasons some aquaculture practices are being frowned upon, as the discharge of untreated effluent could result in eutrophication, leading to excessive algal bloom and destruction of aquatic biodiversity. Various bioremediation options, such as integrated multitrophic aquaculture (IMTA) and aquaponics, have been used to reduce the nutrient load of aquaculture effluent [2,11]. Bioremediation using bacteria is well known; however, mycoremediation, which is the use of fungi in bioremediation is also efficient. The popularity of fungi is increasing due to their potential for application in bioremediation. Fungi species are able to decompose/degrade or modify organic/toxic substances into a less toxic form [129]. They thrive even in environments where bacteria bioremediation is inhibited, such as low pH. The mechanism behind mycoremediation is that fungal biomass/fungal hyphae, in a bid to meet its nutrition and energy requirements, produce bioflocs or beads which capture, absorb and degrade particles in suspension [130]. Fungi have been used for nitrification and denitrification and are known to exhibit a higher capacity of denitrification than bacteria [131]. Fungi have been explored for bioremediation in various industries. They have been used to reduce antibiotics, such as oxytetracycline and ciprofloxacin, in wastewater [132,133]. For example, *Pleurotus ostreatus* was used to degrade oxytetracycline-contaminated soil and water [134], also *Trametes versiocolor* achieved 91% removal of Ciprofloxacin in in vitro experiments [135]. Fungi have been used to degrade numerous medications, such as antidepressants [132], anti-inflammatory drugs [136] and anticancer medications [137], in the pharmaceutical industry. *Trametes versicolor* has also been efficiently used to treat hospital effluents [138]. 

Fungi can also absorb heavy metals present in wastewater; they are able to do this through their cell wall or extracellular polysaccharide slime [19]. Mushrooms, especially the basidiomycetes, are good decomposers, and they secrete extracellular enzymes such as manganese peroxidase, laccase and lignin peroxidases. Polysaccharides derived from mushrooms are known to reduce the toxicity of heavy metals. Jia et al. used polysaccharides from *Ganoderma lucidum* to supplement the diet of common carp (*Caprinus carpio*) reared in cadmium-polluted water [14]. The treatments used were represented by P group (carp reared without polysaccharide supplementation in feed + 0.5 mg/L Cd in water), LG group (carp fed diet containing 2 g/kg polysaccharide + 0.5 mg/L Cd in water) and HG group (carp fed diet containing 2 g/kg polysaccharide + 0.5 mg/L Cd in water), and control (carp reared in unpolluted water and without polysaccharide supplementation in feed) for four weeks. Carp fed *G. lucidum*-supplemented diets all had reduced Cd content in the muscle, blood, liver and brain, the primary target organs in which Cd accumulates. Generally, the muscle tissues of fish exposed to Cd usually have an increased level of total cholesterol, lipids, triglycerides and fatty acids. However, adding *G. lucidum* polysaccharide to the diet of carp led to reduced levels of total cholesterol, lipids, triglycerides and fatty acids in the muscle. The inclusion of *G. lucidum* polysaccharides in diets of fish undid the effect of Cd toxicity such that the FCR of fish fed both high and low doses of *G. lucidum* in their diet and exposed to Cd pollution (LG and HG groups) was found to be significantly lower than those of P groups but was found to be similar to the control groups. Chandra et al. assessed the effectiveness of mycoremediation using mushroom spent bags on fish tanks containing *Ctenopharyngodon idella*, *Labeo rohita* and *Cyprinus carpio haematopterus* fingerlings using various filtration rates [139]. They have reported that mycoremediation using a mushroom spent bag was as efficient as the RAS system.

### 2.8. Bioflocculation

Microalgae are single-celled microorganisms found in aquatic environments. They serve as feed/feed supplements to aquatic animals, thereby minimising the production cost and enhancing aquaculture advancement [140,141]. They are a food and medicine source for humans and serve as bio-fertilizers. They are also valuable for bioremediation of wastewater and biofuel production [35]. Microalgae grow naturally in rivers, lakes and oceans; however, the biomass produced is usually insufficient for large-scale harvesting [142]. Therefore, microalgae are cultivated to meet the required biomass for its intended use. 

One of the bottlenecks associated with microalgae farming is its harvesting. Most microalgae are difficult to harvest due to their relatively small size (2–40 µm) and because they are highly stable in water. Additionally, harvesting microalgae is costly, representing about 20–30% of the total cost of production [143,144]. Various harvesting techniques are used, including sedimentation, coagulation, filtration, flocculation, flotation and centrifugation [145]. Though some of these harvesting methods are helpful, some are time-consuming, toxic to the environment, expensive and require a high energy consumption/utilization. Two or more of these harvesting techniques are sometimes employed to increase the harvesting output. Centrifugation and filtration are widely used for commercial-scale harvesting; however, they require high energy utilization. Coagulation–flocculation is applied in wastewater treatment to remove particles and organic matter because of its easy accessibility and economic technique [146]. The flocculation technique requires the use of flocculants, which could be chemical or natural. Chemical flocculants are frequently employed in sewage treatment, with examples including polyethyleneimine, polyacrylamide, aluminium sulphate and polyaluminium chloride [147]. However, chemical flocculants may not be applicable to microalgal biomass intended for food intended to feed humans and animals. This is because it poses the risk of health and environmental pollution, thus reducing the quality of the microalgal biomass. Hence, a sustainable harvesting technique employing natural flocculants is needed. Natural flocculants are also referred to as bioflocculants, with chitosan as an example. Bioflocculation is a cost-effective and efficient technique for harvesting microalgal biomass because it concentrates algal cells by taking out a substantial amount of water [148,149]. Bioflocculants do not pose the risk of pollution; hence, microalgal biomass harvested with bioflocculants is considered safe for aquatic life and human consumption.

Filamentous fungi are potential bioflocculating agents due to their ability to aggregate and trap microalgae efficiently. Filamentous fungi, such as *A. niger*, *A. oryzae*, *A. lentulus*, *A. fumigatus* and *penicillium* sp., are potential bioflocculants and are helpful in microalgal harvesting. The mechanism through which flocculation is achieved by bioflocculant could be via sweeping, bridging and charge neutralisation [150]. In the application of fungi for microalgal harvesting, fungi spores or pellets are used. Fungi spore-assisted harvesting involves co-cultivating the fungi spores and the microalgae. However, for fungi pellet-assisted harvesting, the pellet is cultured, and active pellets are added into the medium containing the microalgae.

The mycelial pellets of *A. niger* have been used to harvest cyanobacterial biomass due to their high flocculation activity [148,151]. *Aspergillus niger* 7806F3 has been used to flocculate *Synechocystis* biomass. However, the flocculation activity was influenced by the quantity of mycelial pellets and the flocculation time [148]. The higher the mycelial pellets and the flocculation time, the higher the flocculation activity [148]. Harvesting with fungi spores is usually time-consuming, requiring about 24–48 h, when compared with fungi pellets. Ref. [152] has reported that *Chlorella* sp. was harvested with fungi assistance using the spore of *Penicillium* sp. and achieved 99.26% harvesting efficiency within 28 h while a fungi pellet of *Pencillium* sp. assisted harvesting to achieve 98.26 harvesting efficiency within 2.5 h. Ref. [153] tested the efficacy of *Penicillium chrysogenum*, *A. fumigatus* and *A. niger* pellets as a bioflocculants for harvesting the marine microalgae *Nannochloropsis salina* and *Chlorella salina*. All fungi tested showed flocculation activity, but the flocculation efficiency was affected by the flocculation time such that it was higher in 48 h than in 24 h, with an efficiency of 98.9% for the *A. fumigatus* used to harvest *C. salina* (highest efficiency) and 85.9% for the *A. niger* used to harvest *N. salina* (lowest efficiency).

## 3. Limitations on the Use of Fungi

The application of fungi has various benefits; however, some limitations are doubtlessly associated with their use. The effectiveness of fungi in reducing fibre fractions and enhancing nutrient availability is influenced by the duration of fermentation and the dosage administered, which may differ for various feed ingredients, making it difficult for most aquaculture farmers to apply fungi [65]. Therefore, the most effective dosage and fermentation duration for each feed ingredient should be investigated. Most studies on the application of fungi in aquatic diets were conducted in vitro or on a pilot scale under controlled conditions. Therefore, the outcome/result may differ in outdoor/farming conditions.

The various studies available on *Rhizopus* sp. as a flotation agent applied the fungi to pelletised feed, unlike yeast, which is included during feed formulation. As a result, the pellets were found to stick together during fermentation, resulting in fermented pellets of uneven size and shape and which often differ from the initial shape or size. Additionally, fermenting pellets for days may be time consuming [20]. During fermentation, regular stirring of the pellets has been applied to reduce stickiness/clump. This has resulted in minimal success, as the feed still emerges with heterogenous shapes and sizes [20]. This may be avoided by incorporating *Rhizopus* sp. during the feed formulation stage, i.e., before pelleting.

Fungi (*A. niger* and *A. oryzae*) are generally regarded as safe, as they have been used for food production for a long time. However, the major limitation to the use of fungi is the presence of mycotoxins. Some mycotoxins in *A. niger* include Ochratoxin A, Oxalic acid, Fumonisin B_6,_ Fumonisin B_4,_ and Fumonisin B_2_ [154,155,156]. Cyclopiazonic acid and β-Nitropropionic acid have been reported as mycotoxins in *A. oryzae* [157]. The effects of mycotoxins in aquatic animals include weight reduction, poor feed conversion ratio, deterioration in the immune system and high susceptibility to disease [23]. Mycotoxins are carcinogenic to humans; their effect in humans includes inhibition of protein synthesis, inability to produce energy and oxidation stress [158,159]. For example, a concentration of 1000 mg mL^−1^ of *T. asperellum* MT02 was considered best to inhibit vibriosis in vitro. However, on testing for toxicity, *T. asperellum* MT02 incorporated into the feed of Shrimp *L. vannamei* had a toxicity value (LC_50_) at 383.70 mg mL^−1^ due to the presence of mycotoxins, meaning that this concentration led to 50% mortality for *L. vannamei*. The mortality may increase further if the concentration considered best to inhibit vibriosis is applied [40]. Genetic advances are advantageous in addressing mycotoxins in fungi and have been used in deactivating some mycotoxin genes in fungi.

## 4. Conclusions and Recommendations

Fungi have been taken advantage of and explored in the food, chemical and pharmaceutical industries. For aquaculture, the application of fungi is still within the experimentation stage and has barely been explored and adopted. The various studies and examples cited have shown that fungi are advantageous in many aspects of aquaculture, including growth enhancement, increased disease resistance, and increased nutrient utilization, resulting in a reduction of nutrients in aquaculture effluent. Fungi are also applied to improve feed ingredient nutrients and reduce antinutrients and fibre plant protein, making them more suitable for use in aquafeed production. Fungi increase the digestion of plant protein, feed buoyancy and thus aquafeed flotation, and can act as alternatives to synthetic pigments. Fungi promise to be of considerable benefit to the aquaculture industry if their potential is tapped, improving the sustainability and productivity of aquaculture. 

## Figures and Tables

**Table 2:** The application of fungi in the reduction of fibre fractions in fish feed ingredient.

Fibre Fractions	Feed Ingredient	Fungi Species	Raw Ingredient	Fermented	Variation (%)	References
Crude fibre	Rice bran	*Aspergillus niger*	10.66	4.60	56.85	[65]
	Tea dregs	*Trichoderma koningii* *Aspergillus niger*	18.2 18.2	22.3 19.7	−22.53 −8.24	[66]
	Palm kernel cake	*Aspergillus niger*	16.8	14.5	13.69	[16]
	Cotton seed meal	*Aspergillus niger*	30.67	30.57	0.33	[67]
	Sunflower meal	*Aspergillus niger*	20.43	23.05	−12.83	[67]
	Hazelnut meal	*Aspergillus niger*	13.78	8.60	37.59	[67]
	Wheat Bran	*Aspergillus niger*	8.97	5.20%	42.02	[68]
	Brewer’s spent grain	*Aspergillus niger*	13.31%	9.20	30.88	[68]
	Palmkernel cake	*Aspergillus niger*	15.59	14.91%	4.36	[68]
	Wheat bran	*Aspergillus niger* ATCC 200345	10.95	8.97%	18.08	[69]
	Tomato pomace	*Aspergillus niger*	21.71	23.0	−5.94	[70]
	Rice bran	*Aspergillus flavus*	12.05	9.35	22.41	[71]
	Rapeseed meal	*Rhizopus microsporus*	10.8	11.9	−10.19	[72]
ADF	Wheat bran	*Aspergillus niger*	13.46	11.87	11.81	[69]
	Canola meal	*Aspergillus pullulans* (58522) *Aspergillus pullulans* (42023) *Aspergillus pullulans* (Y-2311-1) *Pichia kudriavzevii* *Tricoderma reesei* *Fusarium venenatum* *Mucor circinelloides*	19.9 19.9 19.9 19.9 19.9 19.9 19.9	17.7 18.0 22.4 18.7 19.1 22.5 20.1	11.06 9.56 −12.56 6.03 4.02 13.07 −1	[73]
	Rapeseed meal	*Aspergillus niger*	33.70	30.76	8.72	[15]
	Tomato pomace	*Aspergillus niger*	25.22	26.84	−6.42	[70]
	Wheat bran	*Aspergillus niger* ATCC 52172	13.46	11.87	11.81	[69]
	Hazelnut meal	*Aspergillus niger* ATCC 52172	22.44	19.36	13.73	[67]
	Sunflower meal	*Aspergillus niger* ATCC 52172	23.82	26.15	−9.78	[67]
	Cottonseed meal	*Aspergillus niger* ATCC 52172	39.70	43.13	−8.64	[67]
	Palm kernel cake	*Aspergillus niger*	46.8	35.8	23.50	[16]
	Tea dregs	*Aspergillus niger*	24.2	26.8	−10.74	[66]
	Tea dregs	*Trichoderma koningii*	24.2	29.5	−21.9	[66]
	Rice bran	*Aspergillus niger*	17.28	12.43	28.07	[65]
	Rapeseed meal	*Rhizopus microsporus*	10.8	11.9	−10.1	[72]
NDF	Rapeseed	*Aspergillus niger*	47.08	40.74	13.47	[15]
	Rice bran	*Aspergillus flavus*	12.05	9.35	22.41	[71]
	Tomato pomace	*Aspergillus niger*	21.71	23.00	−5.94	[70]
	Wheat bran	*Aspergillus niger* ATCC 52172	10.95	9.79	10.59	[69]

ADF—acid detergent fibre; NDF-neutral detergent fibre.
